# Draft Genome Sequence of *Agarivorans albus* Strain MKT 106^T^, an Agarolytic Marine Bacterium

**DOI:** 10.1128/genomeA.00367-13

**Published:** 2013-07-18

**Authors:** Motoshige Yasuike, Yoji Nakamura, Wataru Kai, Atushi Fujiwara, Youhei Fukui, Masataka Satomi, Motohiko Sano

**Affiliations:** National Research Institute of Fisheries Science, Fisheries Research Agency, Yokohama, Japan

## Abstract

*Agarivorans albus* is a Gram-negative, strictly aerobic, and agar-hydrolyzing marine bacterium. We present the draft genome sequence of the *A. albus* strain MKT 106^T^, which is composed of 67 contigs (>500 bp) totaling 4,734,285 bp and containing 4,397 coding DNA sequences (CDSs), four rRNAs, and 64 tRNA sequences.

## GENOME ANNOUNCEMENT

Members of the genus *Agarivorans*, which is currently composed of two species, are Gram-negative, strictly aerobic, and agar-hydrolyzing bacteria (1, 2). *Agarivorans albus*, the type species of the genus *Agarivorans*, was isolated by Kurahashi and Yokota (1) from the intestines of healthy marine creatures (molluscs and protochordata) that were collected from the coast of the Kanto area in Japan. Recently, an isolate of *Agarivorans gilvus* taken from a fresh seaweed sample collected from the coast of Weihai in China has been proposed to be a novel member of the genus *Agarivorans* (2). One of the particular phenotypic features of members of the genus *Agarivorans* is their ability to degrade agar (1). This attribute likely plays a role in alga-feeding marine creatures and within the carbon cycle of the marine ecosystem (1, 2). Although several β-agarase genes that catalyze the hydrolysis of agar (reviewed in reference 3) have been identified in *A. albus* (4 –6) and *Agarivorans* sp. (6–11), there is no whole-genome sequence information available for the genus. We therefore undertook to characterize the genomic features of the genus *Agarivorans*, and we present here the draft genome sequence of *A. albus* strain MKT 106^T^ (1).

*A. albus* MKT 106^T^ was purchased from the NITE Biological Resource Center (NBRC), National Institute of Technology and Evaluation (NITE), and whole-genome shotgun sequencing was performed on the Roche 454 GS-FLX+ Titanium sequencing platform. A total of 180,812 reads (63,595,663 bases) were obtained and assembled using Newbler v2.6 (Roche Diagnostics). The assembled genome is composed of 67 contigs (>500 bp) totaling 4,734,285 bp, with a G+C content of 44.2%. The N_50_ contig size is 124,617 bp and the largest contig is 357,826 bp.

The draft genome sequence of *A. albus* MKT 106^T^ was annotated using the Rapid Annotations using Subsystems Technology (RAST) server v4.0 (12) with SEED data sets (13, 14). The draft genome contains 4,397 predicted coding DNA sequences (CDSs) with an average length of 946 bp, four rRNAs (2 5S, 1 16S, and 1 23S), and 64 tRNA sequences. This RAST-based annotation showed the presence of 480 subsystems in the draft genome. A subsystem is a set of functional roles that make up a metabolic pathway, a biological process, a structural complex, or a class of proteins (13, 14). A comparison of genome sequences available in the SEED data sets revealed that *Psychromonas ingrahamii* strain 37 is the closest neighbor of *A. albus* MKT 106^T^ (score, 512), followed by *Shewanella loihica* strain PV-4 (score, 479). Both of these neighbors and *A. albus* belong to the order *Alteromonadales*.

This is the first whole-genome sequence for a member of the genus *Agarivorans*. This genomic information will provide a reference genome data set of *Agarivorans*, as well as help define the phylogenic relationships between this genus and other species of bacteria in future studies.

### Nucleotide sequence accession numbers.

The draft genome sequence of *A. albus* MKT 106^T^ was submitted to DDBJ under accession no. BARX01000001 to BARX01000067.
